# Uncovering differences in the composition and function of phage communities and phage-bacterium interactions in raw soy sauce

**DOI:** 10.3389/fmicb.2023.1328158

**Published:** 2023-12-22

**Authors:** Guiliang Tan, Shaohan Qi, Yi Wang, Xueyan Li, Xiangli Li, Mei Li, Lin Li, Lichao Zhao, Min Hu

**Affiliations:** ^1^School of Material Science and Food Engineering, University of Electronic Science and Technology of China, Zhongshan Institute, Zhongshan, China; ^2^College of Food Science, South China Agricultural University, Guangzhou, Guangdong, China; ^3^School of Health Industry, Zhongshan Torch Polytechnic, Zhongshan, China; ^4^School of Environmental Science and Engineering, Changzhou University, Changzhou, China

**Keywords:** raw soy sauce, phage diversity and functions, phage-host association, metagenome-assembled genome, auxiliary metabolic genes

## Abstract

**Introduction:**

Although the composition and succession of microbial communities in soy sauce fermentation have been well-characterized, the understanding of phage communities in soy sauce remains limited.

**Methods:**

This study determined the diversity, taxonomic composition, and predicted function of phage communities and the phage-host interactions in two types of raw soy sauce (Cantonese-type fermentation, NJ; Japanese-type fermentation, PJ) using shotgun metagenomics.

**Results and discussion:**

These two raw soy sauces showed differences in phage composition (121 viral operational taxonomic units (vOTUs) in NJ and 387 vOTUs in PJ), with a higher abundance of the family *Siphoviridae* (58.50%) in the NJ phage community and a higher abundance of *Myoviridae* (33.01%) in PJ. Auxiliary metabolic functional annotation analyses showed that phages in the raw soy sauces mostly encoded genes with unknown functions (accounting for 66.33% of COG profiles), but the NJ sample contained genes mostly annotated to conventional functions related to carbohydrate metabolism (0.74%) and lipid metabolism (0.84%), while the PJ sample presented a higher level of amino acid metabolism functions (0.12%). Thirty auxiliary metabolism genes (AMGs) were identified in phage genomes, which were associated with carbohydrate utilization, cysteine and methionine metabolism, and aspartic acid biosynthesis for the host. To identify phage-host interactions, 30 host genomes (affiliated with 22 genera) were also recruited from the metagenomic dataset. The phage-host interaction analysis revealed a wide range of phage hosts, for which a total of 57 phage contigs were associated with 17 host genomes, with *Shewanella fodinae* and *Weissella cibaria* infected by the most phages. This study provides a comprehensive understanding of the phage community composition, auxiliary metabolic functions, and interactions with hosts in two different types of raw soy sauce.

## 1 Introduction

A phage is a type of virus that parasitizes prokaryotic microorganisms such as bacteria and archaea. Phages are the most diverse and abundant biological entities on Earth, with a total number of up to 10^31^ particles (10 times the number of bacterial particles), which are widely found in water, soil, animals, the human gut, and fermented foods (Ledormand et al., 2020; Brown et al., 2022). In the fermented food industry, phages are often of great concern due to their ability to cause fermentation failure by lysing the starter cultures (Chen et al., 2016). Currently, numerous phages have been isolated from various fermented foods through traditional culture-dependent methods, such as sauerkraut, natto, fermented cucumber, and wine (Kelsey et al., 2022). Metagenomic sequencing and data are also being used to uncover the composition of the phages in fermented foods (Park et al., 2011; Dugat-Bony et al., 2020; Du et al., 2023). However, these studies have mostly focused on the composition and diversity of phages, while there is limited research on the functional potential of phages in fermented foods (Yu et al., 2022; Du et al., 2023). To date, only one study has reported the interaction between phages and hosts (metagenome-assembled genome, MAG) based on shotgun metagenomics (Queiroz et al., 2023).

Phages, as accessory gene reservoirs, can affect the function of microbial communities through the incorporation and expression of auxiliary metabolic genes (AMGs) in ways that go beyond killing their hosts. AMGs are host-derived genes present in phage genomes that may encode proteins to redirect and enhance the metabolism of the infected host (Hayes et al., 2017). Studies in natural ecosystems have shown that AMGs possess a range of metabolic functions, including antioxidation, carbon metabolism, cell protection, cycling of nutrients, and fatty acid metabolism (Breitbart et al., 2018). Recently, there have also been a small number of reports on the functional potential of phages based on the Clusters of Orthologous Groups of proteins (COG), Kyoto Encyclopedia of Genes and Genomes (KEGG), and CAZymes annotation at the community level in fermented foods (Tamang et al., 2022; You et al., 2022; Du et al., 2023). However, the detailed AMGs contained in a specific phage genome are rarely disclosed (Paillet et al., 2022), which may hamper the full understanding of their auxiliary contributions to microbiome functioning in fermented foods.

Soy sauce, a traditional soy-based fermented condiment, is widely consumed in China and other Asian countries because of its special taste and flavor (Feng et al., 2013; Liu et al., 2015). It was found that the predominant microbial groups in soy sauce fermentation systems included bacterial genera such as *Tetragenococcus, Weissella, Staphylococcus*, and *Bacillus* and fungal genera such as *Aspergillus, Saccharomyces, Candida*, and *Debaryomyces* (Han et al., 2020; Qi et al., 2021; Tan et al., 2022). Compared with the bacterial and fungal communities, there is less research on the phage community during soy sauce fermentation. There are limited reports on the isolation of phages (Uchida and Kanbe, 1993; Wakinaka et al., 2022) and the mechanisms of host–phage interactions, such as specific binding receptors (Wakinaka et al., 2022). The diversity and function of phages as well as phage–host interactions during soy sauce fermentation remain largely unknown. To address this research gap, this work characterized the diversity and composition of phages and further analyzed the phage metabolic functions and phage–host interactions in raw soy sauce with two different types of fermentations [Cantonese-type fermentation (NJ) and Japanese-type (PJ) fermentation] based on shotgun metagenomic sequencing. These results will contribute to a better understanding of the diversity and functions of phages in soy sauce fermentation ecosystems.

## 2 Materials and methods

### 2.1 Sample collection and physicochemical analysis

Raw soy sauce samples during NJ and PJ fermentation were collected from a local soy sauce factory (Pearl River Bridge Biotechnology Co., Ltd.; Zhongshan, Guangdong, China). The samples were collected on day 120 of fermentation. The samples collected from the NJ and PJ fermentation processes were named NJ5 and PJ6, respectively. Triplicate raw soy sauce samples were collected from three different tanks and placed in 50-mL centrifuge tubes and then transported on ice to the laboratory and stored at −20°C until further analysis.

The pH, total acidity (TA), NaCl, and amino acid nitrogen (AAN) contents were analyzed using the titration method with an automatic potentiometric titrator (905-Titrando; Metrohm, Switzerland), as previously described (Tan et al., 2022).

### 2.2 DNA extraction, metagenomic sequencing, and assembly

The total DNA from raw soy sauce samples (0.5 g) was extracted as described previously (Tan et al., 2022). A Qubit 2.0 fluorometer (Invitrogen, Carlsbad, CA, United States) and electrophoresis on a 1% agarose gel were used to determine the DNA quantity and quality. DNA extracted from triplicate samples at the same sampling time were pooled and stored at −20°C for subsequent metagenomic analysis. To obtain metagenomic data, extracted DNA was sheared into ~350-bp fragments using a Covaris M220 nucleic acid shearer (Covaris, Woburn, MA, USA). The total DNA was used for sequencing library preparation with the NEBNext Ultra DNA Library Prep Kit (NEB, Ipswich, MA, USA) and then sequenced on the Illumina NovaSeq 6000 platform (San Diego, CA, USA), leading to 2 × 150 bp, paired-end reads, at the Novogene Bioinformatics Technology (Beijing, China). Raw sequencing data were deposited in the Sequence Read Archive (SRA) of NCBI BioProject under accession number PRJNA1026665.

### 2.3 Identification and classification of phage scaffolds

Adapter sequences were removed from the generated reads, which were then trimmed using Trimmomatic v.0.30 with a quality cutoff of 30, a sliding window of 6 bp, and a minimum length cutoff of 45 bp (Bolger et al., 2014). High-quality reads were assembled using MEGAHIT v1.2.9 (Li et al., 2015). Contigs ≥ 2.5 kb from metagenome assemblies were used to recover phage sequences. For each phage population, the open reading frames (ORFs) were predicted using Prodigal v2.6.3 (Hyatt et al., 2010) with the parameters set as “-p meta -g 11 -f gff -q -m -c.” Phage sequences were identified using VirSorter v1.0.6 (Guo et al., 2021) with default settings and Vibrant v1.2.1 (Kieft et al., 2020). CheckV v1.0.1 was used to assess the completeness, contamination, and quality of phage genomes represented by viral scaffolds (Nayfach et al., 2021). The candidate phages were then clustered based on 95% average nucleotide identity (ANI) over 80% of the sequence length, generating 397 species-level phage populations (viral operational taxonomic units, vOTUs). Phylogenetic trees were constructed using MEGA X software (Kumar et al., 2018) by comparing terminase large subunit (*TerL*) amino acid sequences with viral reference sequences and NCBI-NR by BLASTP. The resulting trees were visualized using iTOL (Letunic and Bork, 2019). The alpha-diversity indices of phage communities were calculated using QIIME2.

### 2.4 Functional annotations and identification of AMGs

The predicted ORFs of phage genomes were annotated by searching against eggNOG v5.0.0 (Huerta-Cepas et al., 2019) using emapper v2.1.12 (Huerta-Cepas et al., 2017) and against KEGG (Kanehisa and Goto, 2000) using KofamScan v1.3.0 (Aramaki et al., 2020). The phage genomes were then subjected to AMG identification and genome annotation using the DRAM-v module from DRAM v1.4.6 (Shaffer et al., 2020). The vOTUs were searched for AMGs using the UniRef90 database and other default databases. The DRAM output contained only putative AMGs; no viral flag (F), transposon flag (T), viral-like peptidase (P), or attachment flag (A) wer included, and all putative AMGs without a gene ID or gene description were also excluded. Gene arrow maps in the AMG-carrying phages were drawn using the R package gggenes (https://cran.r-project.org/web/packages/gggenes).

### 2.5 Reconstruction of host genomes

After the high-quality reads were assembled, as described previously, the assembled-contig coverage was calculated by mapping the initial reads to the contigs larger than 1 kb using the Bowtie2 software (Langmead and Salzberg, 2012). Contigs with a short length and low coverage (length < 1 kb and average coverage < 5) were discarded before the next step of binning. MetaWRAP (Uritskiy et al., 2018) was used to bin the contigs of each sample into MAGs. The qualities of MAGs (completeness and contamination) were assessed with CheckM software (Parks et al., 2015). MAGs with low quality (50% completeness and/or 10% contamination) were removed from downstream analysis. The ORFs of all MAGs were determined using Prodigal (Hyatt et al., 2010). Then, the predicted ORFs of each MAG were checked based on sequence similarity to the NCBI-nr database using BLASTP, with a maximum allowed E-value of 1 e^−10^. To obtain the species-level taxonomic annotation of each MAG, the BLASTP results were parsed using MEGAN (Huson et al., 2007). The MAG taxonomies were investigated using GTDB-Tk programs (Chaumeil et al., 2020). The ANI of MAGs to reference genomes was calculated using FastANI (Jain et al., 2018). Putatively novel MAGs were assigned as potentially novel species when the ANI output obtained using GTDB-Tk was < 95% (Leech et al., 2020).

### 2.6 Phage–host linkage prediction

A sequence alignment-based method (nucleotide–nucleotide) was used to link phage genomes to their hosts *in silico* (Cheng et al., 2022). All phage genomes were compared to the recovered bacterial genomes (MAGs) using BLASTN with the parameters of alignment length ≥2.5 kb, E-value ≤ 10^−3^, bit score ≥ 50, and identity ≥ 70% as previously suggested (Gao et al., 2022). Co-occurrence network analysis and visualization were performed using the interactive platform Gephi v0.9.2 software.

## 3 Results and discussion

### 3.1 Physicochemical characteristics of raw soy sauces

Two samples showed similar physicochemical properties. The pH in NJ5 and PJ6 was 5.07 ± 0.04 and 5.03 ± 0.07, respectively. Also, NaCl (15.00 ± 1.45 g/100 mL in NJ5 and 14.48 ± 0.67 g/100 mL in PJ6) and AAN (0.91 ± 0.02 g/100 mL in NJ5; 0.94 ± 0.01 g/100 mL in PJ6) had similar contents. The TA contents in these two samples were slightly different, with PJ6 containing a higher TA concentration (1.83 ± 0.10 g/100 mL) than NJ5 (1.41 ± 0.02 g/100 mL).

### 3.2 Metagenomic analysis of phage communities

**Figure 1:**
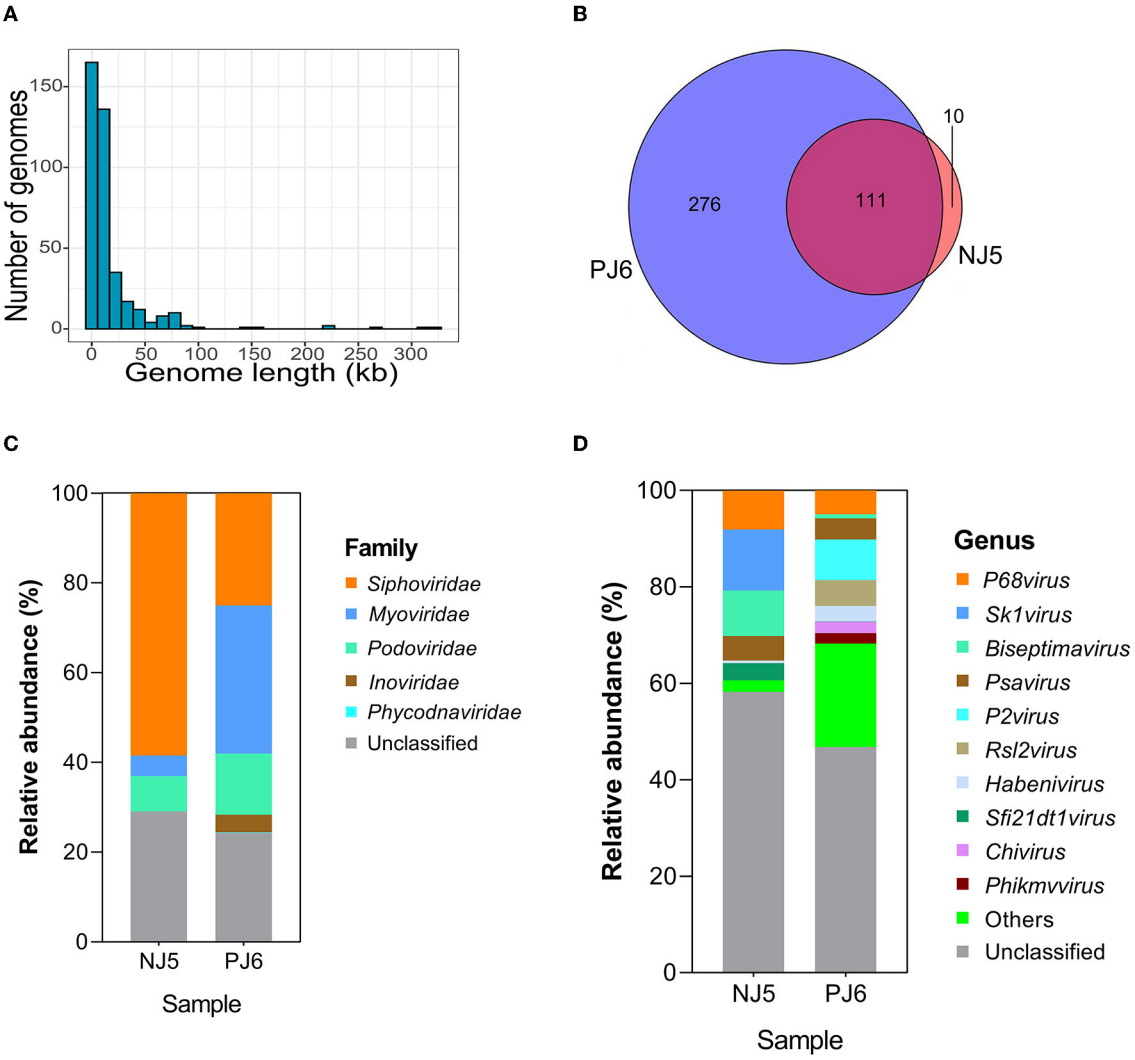
Genome size **(A)**, Venn diagram **(B)**, and taxonomic composition of phages at the family level **(C)** and genus level **(D)** in raw soy sauce. The Venn diagram illustrates the unique and shared viral operational taxonomic units (vOTUs) in the two samples. The 10 most abundant genera are shown. “Others” comprise the less-abundant genera. Sequences that could not be matched to any known taxonomic groups are designated as “unclassified” at the family and genus levels. NJ, Cantonese-type fermentation; PJ, Japanese-type fermentation.

**Figure 2:**
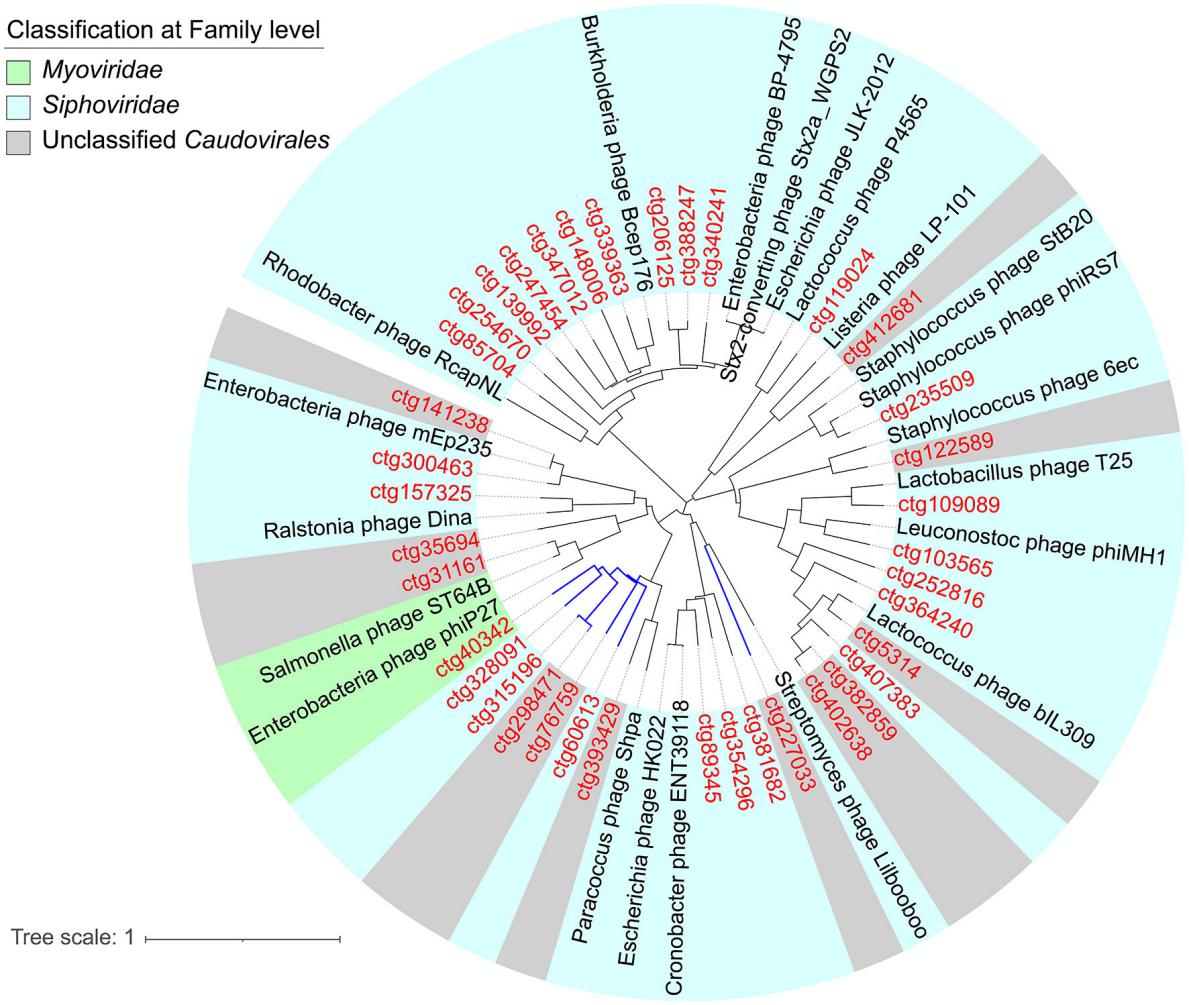
Maximum-likelihood tree of the terminase large subunit protein (*terL*) of *Caudovirales*. Contigs of NJ and PJ raw soy sauce are colored in red. Novel phages are highlighted with blue branches. Reference sequences are colored in black. NJ, Cantonese-type fermentation; PJ, Japanese-type fermentation.

The sequencing generated 70,849,140 sequences from NJ5 and PJ6 samples, with 10.2 and 9.8 Gbp of sequencing data, respectively (Supplementary Table 1). A length of 2.5 kb was selected as the minimum standard for phage genome size, and finally, 397 contigs were found with sizes ranging from 2,505 to 324,623 bp, including five complete phage genomes and six high-quality phage genomes (Supplementary Table 2). The results showed that 92.44% of contigs had lengths < 50 kb, 4.28% (20 contigs) had lengths ranging from 50 to 100 kb, and only 0.75% (three contigs) had lengths >200 kb (Figure 1A). The size of the largest contig was 324 kb, which was a jumbo phage with a complete genome sequence. Among the 397 phages (vOTUs) detected, 111 vOTUs (28.0%) were present simultaneously in the NJ and PJ samples (Figure 1B). PJ, however, harbored a greater number of unique phages (276 out of 397, 69.5% of total phage genomes) than NJ, suggesting a high difference of phages between these two fermentation types. This work then determined the alpha diversity of the raw soy sauce phage communities and found that the Shannon index showed significant differences in phage species richness between the two groups (Supplementary Figure 1). In addition, the prophages present in the two samples had different abundances (35% in NJ and 13.7% in PJ; Supplementary Figure 2). At the family level, *Siphoviridae* (58.5%) dominated NJ, followed by *Podoviridae* (7.87%) and *Myoviridae* (4.55%), while *Myoviridae* (33.01%) and *Siphoviridae* (25.05%) were predominant families in PJ, followed by *Podoviridae* (13.63%) and *Inoviridae* (3.9%; Figure 1C). The majority of the phages in both soy sauce fermentation samples were assigned to the *Siphoviridae, Myoviridae*, and *Podoviridae*, belonging to tailed dsDNA *Caudovirales*. This finding was in agreement with other reports on fermented foods, such as kinema, koumiss, and kimchi (Jung et al., 2018; Kumar et al., 2019; You et al., 2022). However, the abundance of phages in the family *Inoviridae* in PJ was remarkably higher than that in NJ, indicating the preference of these phages for particular soy sauce fermentation environments. The *Inoviridae* phages are characterized by circular ssDNA and long and thin filamentous shapes (Ishnaiwer and Al-Razem, 2013) and are usually detected at low abundances in fermented foods (Du et al., 2023). At the genus level, the two samples (NJ and PJ) exhibited different compositions, but both contained high abundances of *P68virus* and *Psavirus* (8.08 and 5.09% in NJ, respectively; 4.95 and 4.4% in PJ, respectively; Figure 1D). The relative abundances of *Sk1virus, Biseptimavirus*, and *Sfi21dt1* in NJ were higher than those in PJ, while *P2virus* and *Habenivirus* showed the opposite trend. In addition, *Chivirus* and *Phikmvvirus* were only detected in PJ. Subsequently, a phylogenetic tree of phages was constructed based on the *Terl* gene (Figure 2). A total of 38 TerL amino acid sequences were retrieved from contigs and used in the phylogenetic analysis. Seven novel phage contigs were discovered, one of which belonged to *Myoviridae* (ctg40342), three to *Siphoviridae* (ctg328091, ctg315196, and ctg60613), and three to unclassified *Caudovirales* (ctg298471, ctg76759, and ctg227033). The difference in the abundance of phages may be related to the relative abundance and community structure of their hosts and fermentation environmental parameters (such as temperature, oxygen availability, photoperiod, and salinity), which are different between these two soy sauce fermentation processes (Tan et al., 2022). Moreover, high abundances of unclassified phage genera (58.22% in NJ and 46.79% in PJ) were detected in the two samples, indicating that these phages are potentially novel viral species.

### 3.3 Potential functions of the phage community

**Figure 3:**
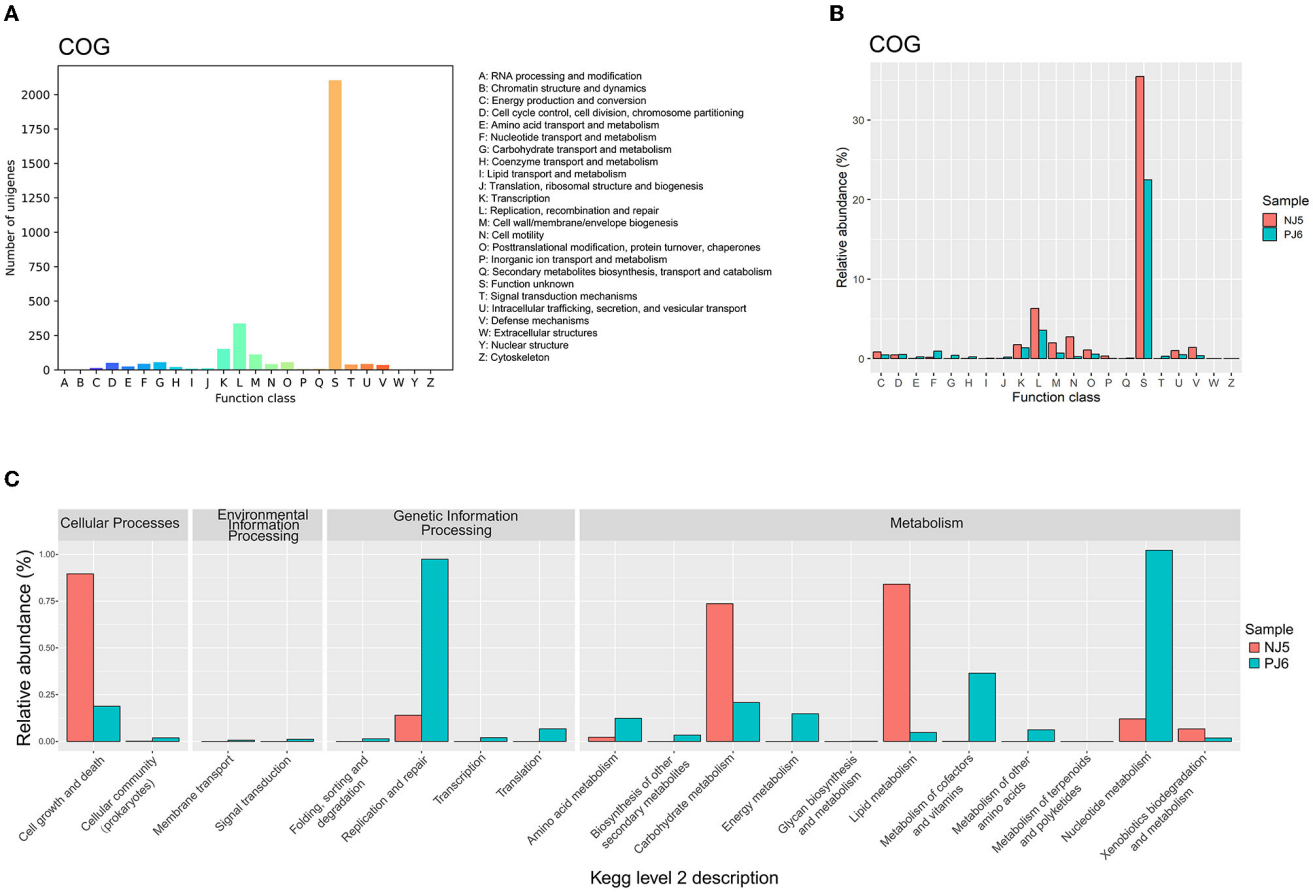
Annotated metabolic functions of phages belonging to the Clusters of Orthologous Groups of proteins [COG; **(A, B)**] and Kyoto Encyclopedia of Genes and Genomes (KEGG) functional classes **(C)** in the two different types of raw soy sauce. The COG and KEGG functional classes were annotated with the eggNOG v5.0 and KEGG databases, respectively. The number of unigenes in COG annotation was summarized using the number of phage open reading frame (ORF) hits. The relative abundances of COG and KEGG functions were determined using the ORF hits to each function class to the total number of ORFs. NJ, Cantonese-type fermentation; PJ, Japanese-type fermentation.

To clarify the potential function of phages in soy sauce fermentation, this study first compared phage sequences with the eggNOG database to obtain the COG function classes. A total of 4,902 ORFs in phage sequences were finally identified. Among these ORFs, 3,172 ORFs (64.71%) were assigned to COG functions. These annotated ORFs were distributed in almost all COG functional categories (Figure 3A). The ORFs obtained were mainly clustered in “replication, recombination, and repair” (L), “transcription” (K), “cell wall/membrane/envelope biology” (M), “posttranslational modification, protein turnover, chaperones” (O), and “cell cycle control, cell division, chromosome partitioning” (D) (Supplementary Figure 3A). In addition to the conventional viral functions mentioned above, the auxiliary metabolic functions of “carbohydrate transport and metabolism” (G) and “amino acid transport and metabolism” (E) were also detected in raw soy sauce samples (NJ and PJ), indicating that phages may have a significant potential influence on the formation of soy sauce flavor. These auxiliary metabolic functions have also been found in viruses from other fermented foods (Yu et al., 2022; Du et al., 2023). During soy sauce fermentation, starch and protein substrates in soybeans are critical flavor precursors (Tan et al., 2022), which are hydrolyzed and converted into monosaccharides, peptides, and amino acids and then metabolized by microorganisms, ultimately contributing to the flavor and quality of soy sauce (Zhou et al., 2022). These carbohydrate- and amino acid metabolism-related ARGs harbored by phages may facilitate their hosts to utilize carbohydrate and biosynthesize amino acids during soy sauce fermentation. However, most COG functions, such as “replication, recombination, and repair” (L), “cell wall/membrane/envelope biogenesis” (M), and “cell motility” (N), along with “function unknown” (S), exhibited higher abundances in NJ than in PJ (Figure 3B).

KEGG functional annotations of the phage community were performed after mapping metagenomic ORFs against KEGG databases. A total of four functional categories in KEGG level 2 were found (Supplementary Figure 3), including cellular processes, environmental information processing, genetic information processing, and metabolism. Among them, metabolic and genetic information processing was the most abundant function. However, functional differences were found at KEGG level 2 between the two samples (Figure 3C). In detail, the NJ sample exhibited a higher relative abundance of genes relating to functions in “carbohydrate metabolism,” “lipid metabolism,” and “cell growth and death,” while PJ exhibited a higher abundance of genes relating to “nucleotide metabolism,” “metabolism of cofactors and vitamins,” “amino acid metabolism,” and “replication and repair.” The higher functional potential of amino acid metabolism identified in PJ was consistent with its COG annotation result.

**Figure 4:**
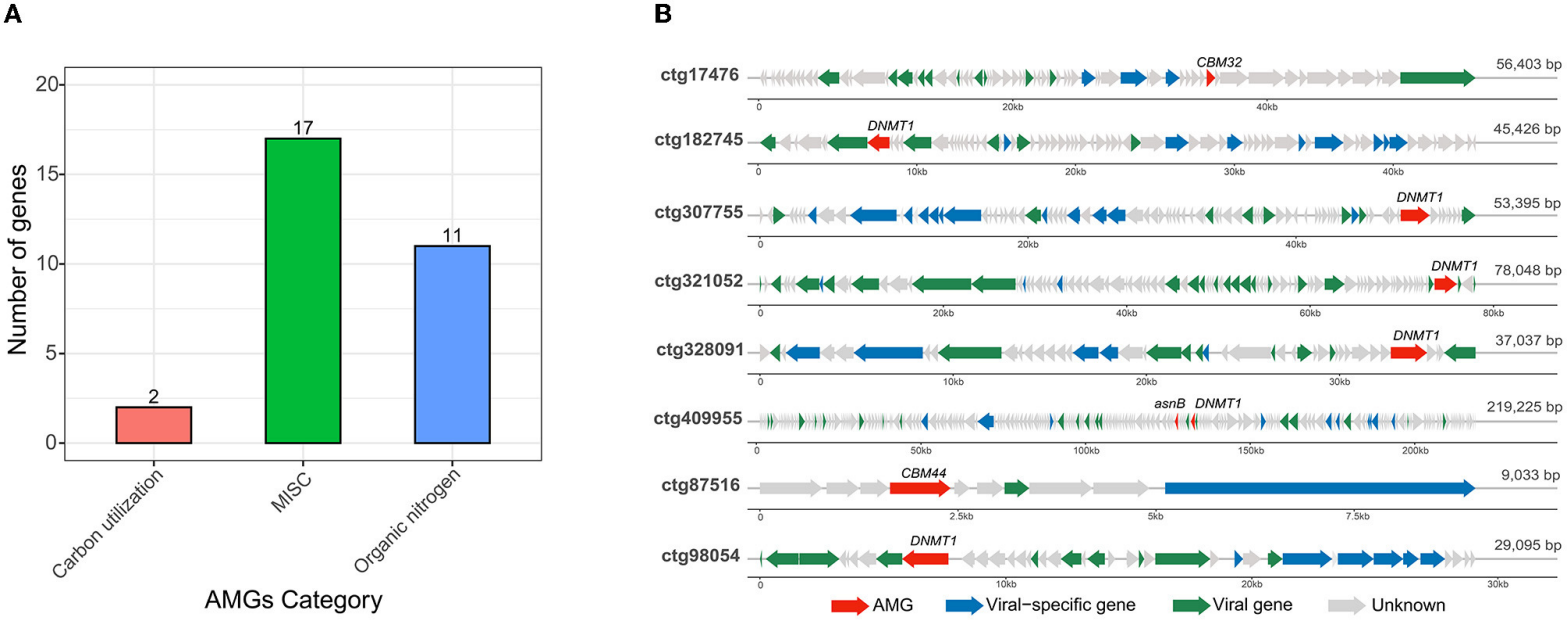
Potential auxiliary metabolic genes [AMGs; **(A)**] and genome map of eight phages containing AMGs **(B)**. The AMGs were identified using DRAM-v annotation. Phage genome maps contain the AMGs involved in carbon utilization and organic nitrogen metabolism. AMGs are shown in red; viral-specific genes (hallmark genes) are shown in blue; known viral genes related to replication, recombination, and repair, cellular processes and signaling, and metabolism are shown in green; and unknown genes are marked in gray.

After illustrating community-wide functional profiles, this work further identified the phage-encoded AMGs that were capable of modifying host metabolism during infection. Thirty AMGs were detected from 25 predicted phages, of which two AMGs (6.6%) were related to the carbohydrate-utilizing category (involved in carbohydrate incorporation), 17 (56.7%) to the MISC category (involved in pyrimidine deoxyribonucleotide biosynthesis), and 11 (36.7%) to the organic nitrogen category (involved in methionine degradation; Figure 4A). The miscellaneous function (MISC) identified in raw soy sauce was related to four enzymes: dUTP pyrophosphatase, dTMP kinase, thymidylate synthase, and dUTP pyrophosphatase, which have not been reported in fermented food systems. The phages with AMGs were selected for subsequent analysis (Figure 4B). Two phages (ctg17476 and ctg87516) that harbored *CBM32* and *CBM44*, belonging to the carbohydrate-binding module (CBM), which is important in carbohydrate metabolism, were found. The CBM family was also identified in isolated Vinitor phages from wine fermentation (vintage; Philippe et al., 2021). Six phages (ctg182745, ctg307755, ctg321052, ctg328091, ctg409955, and ctg98054) were found to possess gene *DNMT1* encoding DNA (cytosine-5)-methyltransferase 1 (EC:2.1.1.37), which was involved in organic nitrogen metabolism (cysteine and methionine metabolism). The gene *DNMT1* was also detected in phage genomes in drinking water distribution systems (Huang et al., 2023). In addition, one contig (ctg409955) was found to carry the gene *asnB* (Figure 4B), which is also associated with organic nitrogen metabolism. The gene *asnB* encodes glutamine-dependent asparagine synthetase (EC:6.3.5.4), also known as L-asparaginase II, an enzyme that converts L-asparagine to aspartic acid and ammonia (Baral et al., 2021) that is involved in alanine, aspartate, and glutamate metabolism.

### 3.4 Phylogenetics of MAGs reconstructed from the raw soy sauce metagenome

To reveal interactions between phages and hosts, this work reconstructed the host genomes based on metagenomic binning. This study obtained 30 stain-level MAGs with at least 50% completeness and < 10% contamination (Supplementary Table 3), of which eight were characterized by high quality (>90% completeness and < 5% contamination). These recruited MAGs were assigned to 22 genera, with the genus *Corynebacterium* harboring the most MAGs (four MAGs), followed by *Weissella* (three MAGs). Many species were recovered, including *Weissella cibaria* (MAG21), *Lactococcus lactis* (MAG28), and *Tetragenococcus halophilus* (MAG26), which are usually detected in the fermentation of soy sauce (Devanthi and Gkatzionis, 2019) or other foods (Xie et al., 2018). In addition, six novel MAGs belonging to six different genera were found (Table 1). Some unexpected species, including *Azospira inquinata* (MAG25), *Shewanella fodinae* (MAG11), and *Sediminibacterium* sp. RGIG5603 (MAG19), were also reconstructed. Overall, the majority of species belonging to the dominant genus in the soy sauce fermentation system (Tan et al., 2022) were reconstructed. Some low-abundance genera such as *Stenotrophomonas, Enterobacter, Lactobacillus*, and *Citrobacter*, were not recovered by metagenomic binning, although they were identified within the TOP15 genera by metagenomic seqencing in this study (data not shown), indicating that binning is more likely to reconstruct species with high abundance.

**Table 1:** Putatively novel species with FastANI identity analysis.

**MAG^a^**	**Predicted name**	**Closest NCBI match^b^**	**Identity (%)**	**Closest NCBI Id**	**Completeness (%)**	**Contamination (%)**
MAG9	*Comamonas* sp.	*Comamonas testosteroni* T5-67	85.81	GCF_014076415.1	84.65	2.69
MAG27	*Cupriavidus* sp.	*Cupriavidus* sp. EM10	87.48	GCF_018729255.1	84.21	4.67
MAG23	*Luteibacter* sp.	*Luteibacter* sp. UNC138MFCol5.1	85.19	GCF_900110505.1	92.74	3.68
MAG10	*Massilia* sp.	*Massilia* sp. Root133	90.45	GCF_001426525.1	81.82	1.13
MAG4	*Mitsuaria* sp.	*Mitsuaria* sp. PDC51	86.71	GCF_900113225.1	90.68	6.29
MAG32	*Sphingomonas* sp.	*Sphingomonas trueperi* DSM 7225	81.84	GCF_011927635.1	85.25	4.60

^a^Only MAGs with completeness >80% and contamination < 10% have been included.

^b^Based on PhyloPhlAn and MEGAN output.

### 3.5 Interaction between phage community and host in raw soy sauce

**Figure 5:**
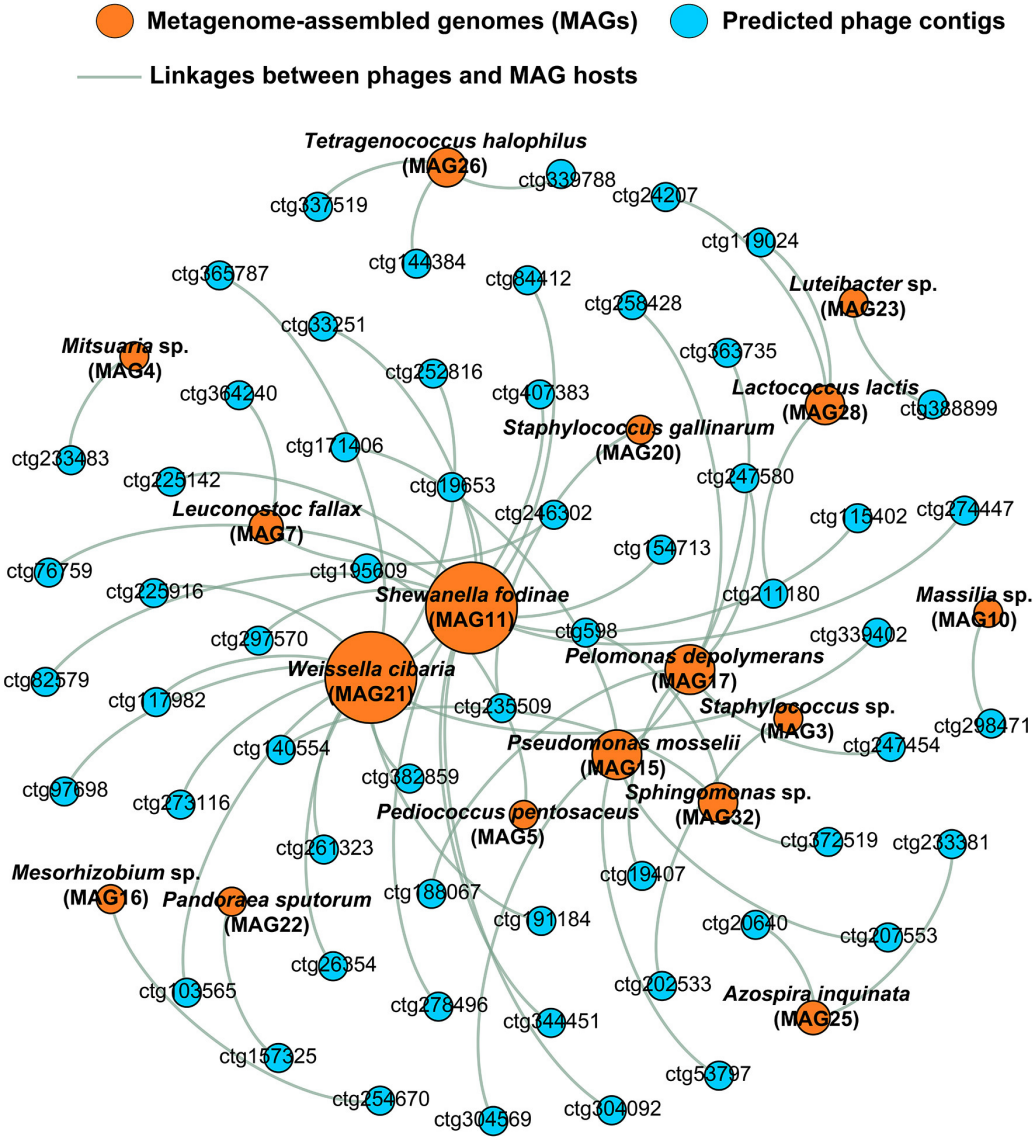
Significant interactions between phages and hosts in raw soy sauce. Bacterial hosts (metagenome-assembled genomes, MAGs) were recovered via shotgun metagenomic sequencing. Phage–host linkages were obtained using sequence alignment-based methods (nucleotide–nucleotide) by comparing the phage genome with the MAG using BLASTN. Phages are shown in blue; bacterial hosts are shown in orange.

Phage–host interactions are crucial for understanding microbial ecology and function. The genome sequence alignment-based method (nucleotide–nucleotide) is one of the important methods used to predict the hosts of uncultivated phages (Gao et al., 2022). To our knowledge, this is the first report to predict the interaction between MAGs and phage genomes in soy sauce fermentation systems. Based on nucleotide sequence homology matches, 57 phage contigs were predicted that were associated with 17 MAGs (Figure 5; Supplementary Table 4), among which *Shewanella fodinae* (MAG11) and *Weissella cibaria* (MAG21) were the most phage-infected bacteria (each infected by 13 phages), followed by *Pseudomonas mosselii* (MAG15) and *Roseateles* sp. (MAG17; both infected by five phages). It is worth mentioning that besides *Weissella cibaria*, some species that were considered to have significant contributions to flavor compounds in soy sauce (Tan et al., 2022), including *Tetragenococcus halophilus* (MAG26), *Lactococcus lactis* (MAG28), *Pediococcus pentosaceus* (MAG5), and *Leuconostoc fallax* (MAG7), had also been infected by phages, harboring 3, 3, 1, and 2 phages, respectively. In particular, the phages of *Tetragenococcus halophilus* detected in this study were found to be phages ctg339788, ctg337519, and ctg144384 (Figure 5), which belonged to *Siphoviridae* and unclassified phages. These results were similar to those of previous reports (Higuchi et al., 1999), in which the *Siphoviridae-*related *Tetragenococcus halophilus* phages ϕ*D-86* and ϕ*D10* were also characterized. For the phage of *Tecogenococcus halophilus*, three phages (ctg339788, ctg144384, and ctg147612,) were found in this bacterium based on CRISPR-spacers match (E-value ≤ 10^−10^; 100% of sequence similarity over the whole spacer), in which phages ctg339788 and ctg144384 were also identified using the nucleotide-nucleotide alignment described above. However, only a very small number of hosts can be predicted based on this CRISPR-spacers match (data not shown). This might be related to the integrity of phages and MAGs (Dikareva et al., 2023). The above results suggested an interaction between a single host and multiple bacteriophages in soy sauce fermentation systems. In addition, it was found that all the phages only infected a single host, suggesting a high degree of specificity that was consistent with phages in other habitats (Achudhan et al., 2023). Here, we speculate that these phage–host relationships influence fermentation dynamics and ultimately the flavor profile of soy sauce through infecting key flavor-producing species such as *Tetragenococcus halophilus, Weissella*, and *Staphylococcus*.

## 4 Conclusion

In this study, the phage communities and phage–host interactions in raw soy sauce samples obtained from two different types of fermentation technology were investigated. The phage composition varied greatly across different fermentation processes, with over half of phage genomes detected in only the PJ sample. The comprehensive analysis of metabolic function revealed that phages encoded a large number of genes with unknown functions, and phages in the NJ sample encoded more AMGs for carbohydrate metabolism and lipid metabolism. Specifically, a large number of flavor-related bacterial species were identified as phage hosts, including *Weissella cibaria, Tetragenococcus halophilus*, and *Pediococcus pentosaceus*, suggesting a high level of interaction among phages and bacteria. Overall, this study provides the first preliminary characterization of the phage community diversity and function and phage–host interactions in soy sauce. Future work is needed to better understand phage adaptability to abiotic factors, co-evolution between hosts and their phages, and how phages affect the bacterial community and flavor characteristics during the entire soy sauce fermentation process, which are critical for understanding phage–host–environment interactions in these particular high-salt niches.

## Data availability statement

The datasets presented in this study can be found in online repositories. The names of the repository/repositories and accession number(s) can be found at: https://www.ncbi.nlm.nih.gov/, PRJNA1026665.

## Author contributions

GT: Project administration, Writing—original draft, Writing—review & editing, Funding acquisition, Methodology. SQ: Methodology, Writing—original draft. YW: Formal analysis, Writing—review & editing. XuL: Investigation, Writing—original draft. XiL: Software, Writing—original draft. ML: Validation, Writing—original draft. LL: Formal analysis, Writing—original draft. LZ: Resources, Writing—original draft. MH: Project administration, Writing—original draft, Writing—review & editing.

## Supplementary material

Supplementary Figure 1 Comparison of Shannon **(A)** and Simpson **(B)** alpha diversity of phage communities in two different types of raw soy sauce. NJ, Cantonese-style fermentation; PJ, Japanese-style fermentation.

Supplementary Figure 2 Relative abundances of prophages in phage communities in two different types of raw soy sauce. NJ, Cantonese-style fermentation; PJ, Japanese-style fermentation.

Supplementary Figure 3 Kyoto Encyclopedia of Genes and Genomes (KEGG) metabolic functions of phages in raw soy sauce. Functional classes were determined at the level 2 classification of KEGG annotations using whole shotgun metagenome assembly-derived open reading frames (ORFs).

Supplementary Table 1 Overview of read statistics for the metagenomic sequence dataset.

Supplementary Table 2 Quality summary of predicted phages.

Supplementary Table 3 Characteristics of curated metagenome-assembled genomes (MAGs).

Supplementary Table 4 Phage–host linkages obtained using sequence alignment-based methods by comparing the phage genome with the metagenome-assembled genome (MAG) using BLASTN.
