# Supplementary figures and images for: Uncovering differences in the composition and function of phage communities and phage-bacterium interactions in raw soy sauce
Source: Front Microbiol. 2023 Dec 22;14:1328158. doi: 10.3389/fmicb.2023.1328158 (PMC10766790; doi:10.3389/fmicb.2023.1328158)

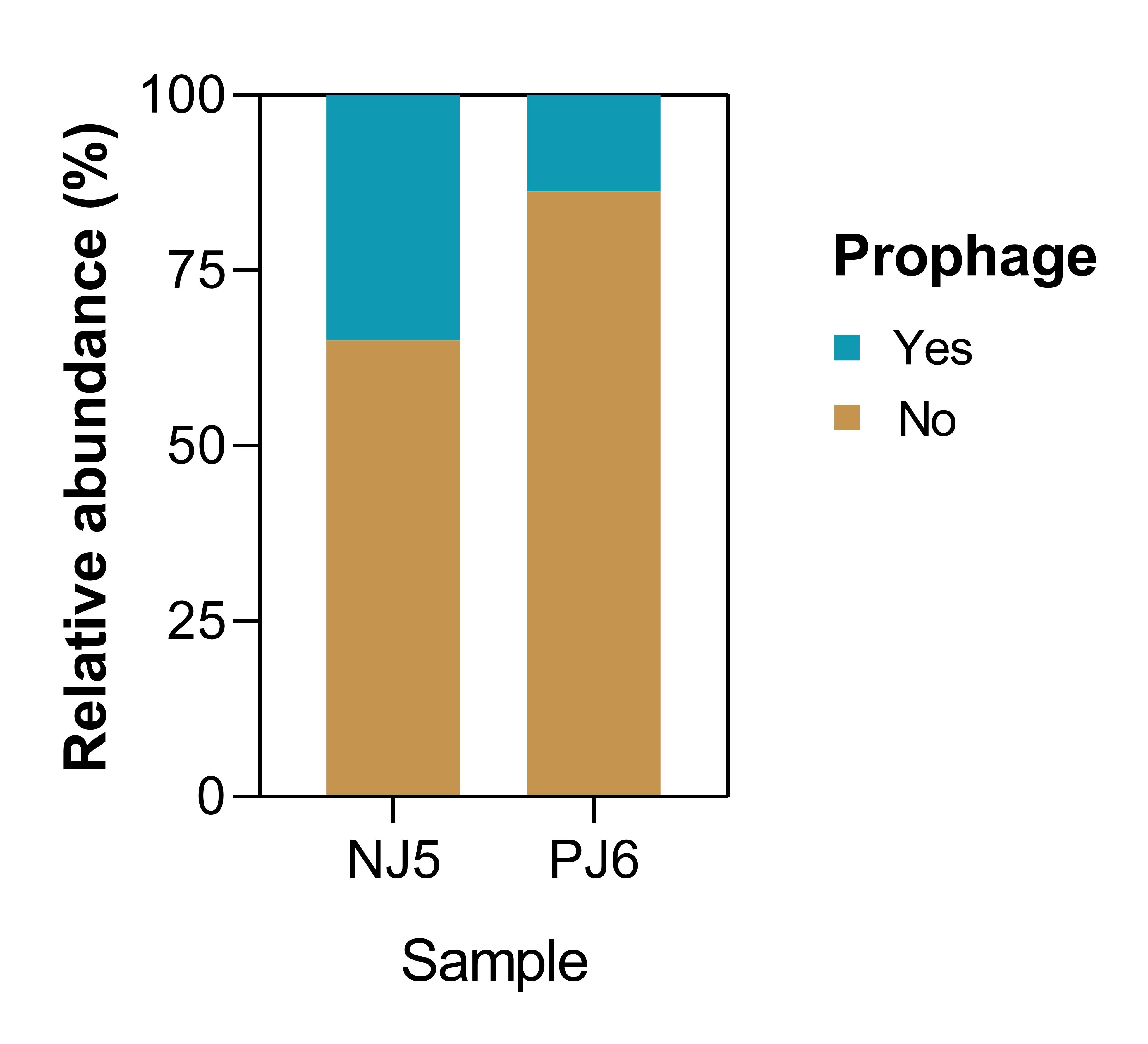

Supplement: Supplementary Figure 2 — Relative abundances of prophages in phage communities in two different types of raw soy sauce. NJ, Cantonese-style fermentation; PJ, Japanese-style fermentation. [file Image_2.JPEG]
